# Interventions to enhance mental health and wellbeing among international college students: A systematic review and meta-analysis protocol

**DOI:** 10.1371/journal.pone.0310645

**Published:** 2024-09-19

**Authors:** Cheng-Ching Liu, Qi Huang, Angela Chia-Chen Chen, Charles Liu, Yuqing Liu

**Affiliations:** 1 College of Nursing, Michigan State University, East Lansing, MI, United States of America; 2 College of Social Science, Department of Human Development and Family Studies, Michigan State University, East Lansing, MI, United States of America; 3 Education and Outreach, Division of Student Life & Engagement. Michigan State University, East Lansing, MI, United States of America; 4 College of Agriculture and Natural Resources. Department of Community Sustainability. Michigan State University, East Lansing, MI, United States of America; Access Alliance Multicultural Health and Community Services: Access Alliance, CANADA

## Abstract

**Background:**

International students contribute significantly to both the economy and the intellectual and cultural landscape of host countries. Their interactions with domestic students foster personal, socioeconomic, and political development, promopting a broader understanding of diverse cultures and values. This highlights how crucial international education is for staying competitive globally. However, international students often face challenges such as poor mental health, linguistic and cultural barriers, acculturative stress, and limited health literacy. Therefore, supporting their academic success and well-being on college campuses is essential. This protocol aims to describe strategies used to evaluate the effect of interventions on international students’ mental health and wellbeing and propose directions for future research based on the evidence.

**Methods:**

We will conduct an extensive search in several databases including CINAHL, PubMed, Web of Science, PsyInFO, ERIC, and Google Scholar with no date limits. Two reviewers will independently screen the literature and extract data. We will then conduct meta-analyses of the extracted data.

**Discussion:**

To the best of our knowledge, this study is the first systematic review with meta-analysis focusing on interventions to enhance mental health and wellbeing among international college students. This study will provide most updated empirical evidence on the effects of interventions aimed to improve international students’ mental health and wellbeing. The findings from this study will summarize the importance of a range of interventions being available to international students who experience psychological distress and the effectiveness of each intervention. This study will also highlight the gap for researchers to focus on for future studies.

**Trial registration:**

**PROSPERO registration number:** CRD42024528767.

## Introduction

United States (U.S.) higher education institutions host approximately one million international students [1], coming from over 227 countries and representing every continent in the world [2]. The majority of these students are enrolled in Student and Exchange Visitor Program (SEVP)-certified associate, bachelor’s, master’s, or doctoral programs, with a significant portion pursuing degrees in STEM fields (i.e. science, technology, engineering and math) and business and management [2]. These international students not only enrich the diversity of educational institutions but also make substantial financial contributions to the host country. For instance, during the 2021–2022 academic year, their contributions amounted to approximately $34 billion to the U.S. economy and supported the creation of over 458,000 jobs [3].

International students contribute significantly to the economy and enhance the intellectual and cultural landscape for domestic students. They bring a wealth of diverse perspectives and skills to our learning environments and workforce. For decades, universities worldwide have benefited from the intellectual, cultural, and educational enrichment provided by international students, who bring a rich variety of experiences and viewpoints to host institutions [4]. For example, these students offer diverse perspectives in the classroom and foster mutual understanding and appreciation of global differences. Embracing international students on U.S. campuses is vital because their contributions positively impact the student population in numerous ways, including enhancing academic prestige, enriching cultural exchange, and boosting financial revenue [4–6]. Interactions between domestic and international students foster enduring effects on personal, socioeconomic, and political development [7]. Increased international student presence on campus presents more chances for domestic students to engage with diverse cultures, prompting them to question and broaden their existing beliefs and values [7]. This underscores the significance of international education as pivotal to U.S. competitiveness in the contemporary global economy and geopolitical context [8]. International students, with their diverse backgrounds and perspectives, play a pivotal role in enriching research endeavors and fostering innovation across various fields [8]. Their unique experiences and cultural insights bring fresh perspectives to scientific and technological challenges, igniting novel approaches and solutions. By collaborating with peers from different parts of the world, international students contribute to the cross-pollination of ideas and methodologies, driving forward scientific and technological advancement [8, 9]. Their presence not only enhances the quality and depth of research but also promotes a global mindset within academic and professional communities, leading to more inclusive and impactful outcomes [9–11]. Therefore, supporting their academic success and wellbeing on college campuses in host countries is imperative.

International students encounter numerous challenges during their academic journeys, with discrimination significantly exacerbating these difficulties [6]. They often deal with acculturative stress [12], academic pressure [13], and loneliness [14], which are compounded by various forms of discrimination, such as alienation, derogatory stereotypes, and hate crimes—issues that have been particularly pronounced during the COVID-19 pandemic [15–19]. This discrimination severely impacts their mental health, leading to higher rates of depression, anxiety, self-harm ideations, and suicide attempts compared to their American peers [16, 17]. The COVID-19 pandemic has further intensified these stressors, worsening issues related to discrimination, financial strain, and cultural adjustment [16]. Recent research studies indicate that the pandemic has had a significant impact on the physical and mental well-being of international students [17–20]. Additionally, findings from the World Mental Health International College Student (WMH-ICS) initiative, which surveyed 13,984 first-year students, highlight a marked reluctance among international students to seek mental health treatment [21]. This reluctance is especially notable among those experiencing major depression, alcohol abuse, or suicidal thoughts and behaviors, as noted by Ebert et al. [21].

Barriers to accessing mental health resources for international students include a preference for alternative support systems, stigma, cultural stigma, and fear of negative consequences [20, 22, 23]. Cultural stigma is significant, as many international students come from backgrounds where mental health issues are heavily stigmatized or seen as personal weaknesses [24]. This discourages seeking help and can lead to reluctance in using mental health services in their host country. Additional barriers arise from cultural differences in perceiving and addressing mental health. In some cultures, openly discussing mental health struggles is uncommon, and there is a strong emphasis on maintaining personal and familial honor [25]. This can deter individuals from seeking help, and fear of being judged by unfamiliar counselors may further exacerbate the problem. Consequently, international students might turn to informal support networks, which may lack the professional expertise needed for serious mental health issues. Despite the high need for psychological support, international students often underutilize counseling services and show high rates of premature drop-out [26, 27]. Research shows that 27.4% experience major depression, 20.0% struggle with generalized anxiety, 17.2% engage in self-injury, and 8.8% have suicidal ideation [26, 27]. These statistics highlight the urgent need for targeted mental health support. Further, many studies indicate that acculturative stress is linked to negative mental health outcomes and risky health behaviors [26, 27]. Specifically, anxiety and depression have been shown to significantly predict binge drinking, drinking and driving, and cigarette smoking among 1,201 international students from 52 U.S. universities [28].

The primary purpose of this paper is to develop a protocol for systematically reviewing interventional studies tailored to the specific needs of international students. By outlining a comprehensive protocol, this protocol aims to describe strategies used to evaluate the effect of interventions on international students’ mental health and wellbeing and propose directions for future research based on the evidence. Considering the diverse range of interventions implemented by colleges and universities worldwide, such as counseling services, peer support programs, or psychoeducational workshops, a structured protocol becomes even more essential to thoroughly assess these interventions’ impact across different contexts. This will not only enhance the quality of research in this field but also contribute to the development of evidence-based strategies for addressing mental health issues within this population.

## Methods

### Objective

This review will synthesize findings from research on the mental health of international students, incorporating quantitative, qualitative, and mixed-method studies. It will be reported according to the Preferred Reporting Items for Systematic Reviews and Meta-Analyses (PRISMA) guidelines to ensure comprehensive and transparent reporting [29]. Through this systematic review and meta-analysis, we aim to identify interventions utilized to alleviate mental health issues, distress, self-criticism, and enhance well-being among international students. Furthermore, we will assess the effectiveness of each intervention included in the analysis.

### Study design

This protocol was registered in the PROSPERO database (International Prospective Register of Systematic Reviews), with registration number CRD42024528767, and is reported in accordance with the guidelines provided in the PRISMA-P guide (Preferred Reporting Items for Systematic Review and Meta-Analyses Protocol) [29]. A systematic review protocol should present the methodological strategies that will be used in the systematic review, such as search strategies, eligibility criteria, what data will be extracted from the selected articles, what the variables of interest are, how the data will be analyzed, and how heterogeneities will be handled [30, 31]. Thus, the protocol should demonstrate transparency in the systematic review process.

### Eligibility criteria

To be included, studies must be evaluated an intervention related to mental health among international students. There are no restrictions on the settings or countries of the interventions, and there are no date limits; these can be in-person or online format or mobile messaging. Educational or medical visits and self-help interventions will be evaluated. A control group is not a necessary requirement for inclusion within this review.

Studies meeting the following criteria will be included: (1) an intervention study with pre-and-post data; (2) targeted international students; (3) aimed to improve mental health or wellbeing; and (4) published in English and peer-reviewed journals. Exclusion criteria covered conference abstracts, published theses or dissertations, letters to editors, curriculum development, published protocols/guidelines, literature reviews, and syllabus material. Short-term exchange students will be excluded. Within the screening process, discrepancies were resolved by an additional reviewer.

### Literature search

The bibliographic databases are CINAHL Complete (EBSCOhost), PubMed, Web of Science, ERIC (ProQuest), PsycINFO, and Google Scholar and focused on 3 areas: 1) international students, 2) mental health interventions, and 3) clinical or educational settings. The search will be modified for each database to include appropriate controlled vocabulary but remained largely similar across databases. For example, the complete search strategy in PubMed was (("international student" OR "international students" OR "foreign student" OR "foreign students)" AND ("substance abuse" OR "binge drinking") AND ("mental health" OR depress* OR anxi* OR "anxiety disorder" OR "anxiety disorders" OR "depressive disorder" OR "depressive disorders" OR "Depression"[Mesh] OR "Depressive Disorder"[Mesh] OR "Mental Health"[Mesh] OR "Anxiety"[Mesh] OR "Anxiety Disorders"[Mesh] OR "well-being" OR wellness OR stress* OR "Stress, Psychological"[Mesh])).

### Screening procedure

All identified articles will be exported to Covidence for further screening. The screening procedure will be carried out in two stages and independently by two reviewers. In the first stage, the first and second authors will independently screen the titles and abstracts of the articles. They will then read the full texts to assess eligibility and determine if the studies meet the inclusion criteria. A third reviewer will decide on the article’s inclusion in case of disagreement. A fourth reviewer will randomly sample 10% of the studies from a pool of articles that have been excluded as a cross-examination. The screening procedure will be done without any contact between the two reviewers in order to avoid influences on the decision process.

### Data extraction

Data extraction will be performed by filling out an Excel sheet with a detailed description of the main information of the selected studies and will also be performed by the first and second authors independently in order to avoid measurement bias, which occurs due to misinterpreting information or even the loss of important data collection. If information is insufficient, corresponding authors of articles may be contacted for clarification or more information.

The following data items will be extracted for each study, when available: (a) study identification items (authors, year of publication), (b) study design characteristics (purpose, sample size, intervention design/type such as survey, interview focus group, any control groups, type of assessment, duration of intervention, length of follow-up assessments), (c) target population items (age, gender), (d) setting (recruitment strategy, online or in-person setting), (e) drop-out rate and (f) outcomes (anxiety, depression, stress, seeking help intention, etc.).

### Quality assessment

In order to evaluate the quality of research and risks of bias, two independent reviewers will assess the quality of each article using the Mixed Methods Appraisal Tool (MMAT) [32]. MMAT is widely used for critical research appraisal and is designed for systematic mixed study reviews. The tool is appropriate for assessing qualitative research, randomized controlled trials, non-randomized studies, quantitative descriptive studies, and mixed methods studies [32–36]. Chosen studies will be rated as having strong, moderate, or weak quality (vs. assigning overall quality scores) as Hong et al. recommended [32].

### Data synthesis

The main outcome to be evaluated is what interventions have been used to improve international students’ mental health and well-being such as anxiety, depression, stress. In addition, other outcomes will also be analyzed, such as the effectiveness of each intervention. A detailed description of the results for all included studies will be provided in text and tables. Characteristics include study design and characteristics including sample size, duration, follow-up period, student population, intervention characteristics (i.e. mindfulness, writing, reading, social support), technical implementation (i.e. online, mobile, or in-person), duration, study and intervention drop-out rate, assessment tool used to determine effectiveness of intervention, strengths and limitations.

### Meta-analysis

We will conduct a quantitative synthesis of the data retrieved from the selected studies using both Stata and R. This will involve employing appropriate meta-analytic techniques, including random-effects modeling, to estimate overall effect sizes and assess heterogeneity across studies [37]. We choose to report Hedge’s *g* instead of Cohen’s *d* for effect size calculation to adjust for small sample bias [38]. In situations where studies reported results of multiple outcomes, we will prioritize and select the outcome measure based on two rules. The primary outcomes in this review are depression and anxiety, while stress is considered a secondary outcome. We will measure changes from baseline using validated assessment tools, without restricting the type of indicator assessment scales. Following the Cochrane Handbook guidelines, we will standardize results across different scales using standardized mean differences (SMDs) with 95% confidence intervals (CIs).

Due to the diverse nature of the design of included studies, we anticipate categorizing them into various design types, including treatment-control, pre-post treatment-control, and treatment group pre-post only designs. This categorization will enable us to employ the appropriate tools and methods for calculating effect sizes accurately. For each study, we will extract key information, including sample sizes, means and standard deviations of the outcome measures being assessed during the pre- and post-test, and pre-post test correlations, if available. This data extraction process will allow us to calculate effect sizes for each study using standardized formulas.

Specifically, for randomized controlled trials (RCTs), we will extract the treatment and control groups’ sample sizes, means and standard deviations from the post-test (calculated using Stata *meta esize*). For non-RCT pre-post treatment-control design, we will extract the sample sizes, means, standard deviations, and pre-post correlations of both the treatment and the control groups from the pre- and post-test (R metafor package *escalc*) [39]. For non-RCT treatment group pre-post only design, we will extract the treatment group sample sizes and means of the pre- and post-test, pretest standard deviation, and the pre and post correlation to calculate its effect sizes [40, 41]. Last, for studies that only reported regression coefficients and other statistical tests as their measures of effect sizes, we will use R package *esc* to convert the reported *beta* or *r* to Hedge’s *g*.

After calculating the effect size for each intervention study, the next step is to aggregate these effect sizes to provide an overall estimate of the intervention’s effectiveness. This aggregation process involves combining the effect sizes using Stata *meta* command. By synthesizing the results across multiple studies, we can derive more robust conclusions about the overall impact of various interventions on international students’ mental health. We will report the overall effect size θ, its confidence interval, and the test statistic *z* and *p*-value, as well as a visual forest plot. We will also examine the presence of heterogeneity between studies using indices τ^2^, I^2^, H^2^ and the chi-squared test of homogeneity.

To address between-study variability, subgroup analyses will be conducted to explore potential sources of heterogeneity and examine whether intervention effectiveness varies across different contexts. These analyses may involve stratifying the data based on intervention type, duration, study setting, design, or participant demographics. Understanding how intervention effects differ across these subgroups will provide insights into the factors influencing the effectiveness of mental health interventions for international students. Given variations in cultural background, language proficiency, acculturation level, and previous experiences, not all international students may respond equally to the same intervention. Identifying which interventions are most effective for specific subgroups and why can guide the development of targeted and culturally sensitive interventions, ultimately enhancing mental health outcomes for international students.

Last, to assess the publication bias that studies with insignificant results tend to be suppressed from being published, we will use the funnel plot and egger test to visually evaluate and statistically test its (a)symmetry. Moreover, we will conduct a trim-and-fill analysis to assess the robustness of our meta-analysis results against potential studies that might be missing from the current pool of publications.

## Discussion

Such a review ensures a structured and rigorous approach to synthesizing existing evidence, thereby enhancing the reliability and validity of the review findings. By systematically searching, appraising, and synthesizing relevant literature, this review enables researchers to identify and analyze trends, patterns, and gaps in the current knowledge landscape.

This review’s inclusion of subgroup analysis is particularly advantageous. By disaggregating data based on various factors such as intervention type, the review can provide more nuanced insights into the effectiveness of different interventions for international students. This approach allows for a comprehensive understanding of which interventions are most beneficial for this demographic, thereby guiding future research and policy efforts more effectively.

However, it is essential to acknowledge the limitations of this review. One notable limitation is the potential language bias, as the review may primarily include studies published in English, thus excluding relevant research published in other languages. Second, this review may face challenges in synthesizing heterogeneous interventions with various outcome measures across studies, which could affect the comparability and interpretability of the findings.

Our findings, despite these limitations, will contribute valuable insights to the currently limited literature on mental health complaints among international students, including depressive symptoms, academic stress, and loneliness, as well as interventions applied to address these issues. This review’s emphasis on transparency, reproducibility, and methodological rigor will significantly advance our understanding of mental health interventions for this population, benefiting both international students and society as a whole.
